# Primary Cortical Cell Tri-Culture-Based Screening of Neuroinflammatory Response in Toll-like Receptor Activation

**DOI:** 10.3390/biomedicines10092122

**Published:** 2022-08-29

**Authors:** Noah Goshi, Hyehyun Kim, Erkin Seker

**Affiliations:** 1 Department of Biomedical Engineering, University of California—Davis, Davis, CA 95616, USA; 2 Department of Electrical and Computer Engineering, University of California—Davis, Davis, CA 95616, USA

**Keywords:** microglia, neuroinflammation, in vitro model, morphology, toll-like receptor, CNS, screening

## Abstract

The activation of toll-like receptors (TLRs) in the central nervous system (CNS) can lead to neuroinflammation and contribute to many neurological disorders, including autoimmune diseases. Cell culture models are powerful tools for studying specific molecular and cellular mechanisms that contribute to these disease states and identifying potential therapeutics. However, most cell culture models have limitations in capturing biologically relevant phenomena, due in part to the non-inclusion of necessary cell types. Neurons, astrocytes, and microglia (critical cell types that play a role in neuroinflammation) all express at least a subset of TLRs. However, the response of each of these cell types to various TLR activation, along with their relative contribution to neuroinflammatory processes, is far from clear. In this study, we demonstrate the screening capabilities of a primary cortical cell tri-culture of neuron, astrocyte, and microglia from neonatal rats. Specifically, we compare the neuroinflammatory response of tri-cultures to that of primary neuron-astrocyte co-cultures to a suite of known TLR agonists. We demonstrate that microglia are required for observation of neurotoxic neuroinflammatory responses, such as increased cell death and apoptosis, in response to TLR2, 3, 4, and 7/8 activation. Additionally, we show that following TLR3 agonist treatment, microglia and astrocytes play opposing roles in the neuroinflammatory response, and that the observed response is dictated by the degree of TLR3 activation. Overall, we demonstrate that microglia play a significant role in the neuroinflammatory response to TLR activation in vitro and, hence, the tri-culture has the potential to serve as a screening platform that better replicates the in vivo responses.

## 1. Introduction

Neuroinflammation plays a significant role in most neurological diseases including multiple sclerosis [1], neurodegenerative diseases [2,3,4], brain injury [5], and stroke [6]. It typically refers to the innate inflammatory response within the central nervous system (CNS), primarily driven by microglia and astrocytes [7]. Following a neuroinflammatory stimulus, microglia and astrocytes become “activated” and can be characterized by changes to their morphology and phenotype [8,9,10]. Depending on a multitude of factors, this response can take on a variety of roles, ranging from rescuing damaged neurons and promoting recovery to increasing pro-inflammatory cytokine expression and increasing cell death [6]. Additionally, it has been shown that cellular cross-talk between neurons and glial cells, some of which require direct cell-cell interaction, plays a significant role in the observed neuroinflammatory response [11,12]. While there has been much progress in determining the cellular and molecular mechanisms driving these disparate outcomes of neuroinflammation, due to the high complexity and interconnectivity, there is still much that is unknown.

Toll-like receptors (TLRs) are an evolutionarily conserved class of pattern-recognition receptors (PRRs) that recognize a multitude of pathogen-associated molecular patterns (PAMPs) produced by bacteria, viruses, and fungi, and are significantly involved in the innate immune response [13]. Additionally, TLRs recognize endogenous damage-associated molecular patterns (DAMPs) such as extracellular matrix components, high-mobility group box protein 1 (HMGB1), and mitochondrial DNA [14]. While TLR expression is primarily associated with macrophages, neutrophils, and other cells associated with the innate immune system, TLRs are expressed in many other cell types, including both neurons and glial cells in the CNS [15,16]. Additionally, CNS-specific factors such as Aβ_1–42_, along with DAMPs from damaged or necrotic neuronal cells, have been shown to activate TLRs on both neurons and glial cells, leading to a robust neuroinflammatory response [17,18]. However, the exact nature of the neuroinflammatory response to the activation of different TLRs has been debated. This can in part be attributed in part to the limitations of in vitro methodologies, which are often confined to the culturing of a single cell type. For example, in one study, astrocyte cultures spiked with the TLR3 ligand poly (I:C) showed increased gene expression for many neurotrophic factors, and the conditioned media promoted neuron survival in an organotypic culture [19]. Conversely, a similar study conducted on isolated microglia showed a significant increase in the secretion of pro-inflammatory factors such as TNF-α and IL-6 [20]. Therefore, there is a need to better understand the relative contributions of different CNS cells on the observed neuroinflammatory response to TLR activation.

In this study, we use a newly developed neuron, astrocyte, and microglia tri-culture model that better mimics the in vivo response to a number of neuroinflammatory stimuli [21]. The tri-culture is produced by taking primary cortical cells from neonatal rats and maintaining them in a serum-free culture medium that was specifically designed to support all three cell types. Compared to other mixed-culture models that require the separate maintenance and addition of cells at specific time points, the relative simplicity of this tri-culture model makes it well suited for high-throughput experiments, and an effective model for the early screening of therapeutic molecules. Here, we investigate the response of the tri-culture and neuron–astrocyte co-cultures to a suite of TLR agonists, and quantify the responses of both cultures using a number of known neuroinflammatory markers including changes in cell death, apoptosis, and astrocyte and microglia morphology. We observed significant differences in the neuroinflammatory response between the co- and tri-cultures to nearly all tested TLR agonists, highlighting the role of microglia in neuroinflammatory processes. Additionally, we observed changes in microglia morphology in response to the activation of all tested TLRs (1–9), suggesting that rat microglia express TLR1–9, in agreement with previous studies on mouse [22] and human [20] microglia TLR expression. The results highlight the influence of cell culture composition on the neuroinflammatory response to different TLR agonists, and emphasize that microglia are required in order to observe many of the neurotoxic aspects of neuroinflammation in response to TLR activation found in vivo.

## 2. Methods

### 2.1. Primary Cortical Culture

All media were prepared as previously described [21,23]. Briefly, the plating medium consisted of Neurobasal A culture medium supplemented with 2% B27 supplement, 1× Glutamax, 10% heat-inactivated horse serum, and 20 mM HEPES at pH 7.5, while the co-culture medium consisted of Neurobasal A culture medium supplemented with 2% B27 supplement and 1× GlutaMAX (all from ThermoFisher, Waltham, MA, USA). The tri-culture medium consisted of supplementing the co-culture medium with 100 ng/mL mouse IL-34 (R&D Systems, Minneapolis, MN, USA), 2 ng/mL TGF-β (Peprotech, Cranbury, NJ, USA), and 1.5 μg/mL ovine wool cholesterol (Avanti Polar Lipids, Alabaster, AL, USA). Due to the limited shelf life of IL-34 and TGF-β, the tri-culture medium was made fresh each week.

The cells were cultured on tissue culture-treated polystyrene plates (ThermoFisher) or glass bottom chambered coverslips (ibidi, Fitchburg, WI, USA), which were pre-treated by coating the substrates with 0.5 mg/mL of poly-L-lysine (Sigma, Burlington, MA, USA) in B-buffer (3.1 mg/mL boric acid and 4.75 mg/mL borax, Sigma) for 4 h at 37 °C and 5% CO_2_, washing them with sterile deionized (DI) water, and finally covering them with the plating medium.

All procedures involving animals were conducted in accordance with the National Institutes of Health Guide for the Care and Use of Laboratory Animals, following protocols approved by the University of California, Davis Institutional Animal Care and Use Committee. Timed-pregnant Sprague–Dawley rats were purchased from Charles River Laboratory (Hollister, CA, USA). All animals were housed in clear plastic shoebox cages containing corncob bedding under a consistent temperature (22 ± 2 °C) and 12 h light-dark cycle. Food and water were provided ad libitum. Primary cortical cultures were prepared from perinatal day 0 rat pups as previously described [24]. Neocortices from all pups in the litter were pooled, dissociated, and plated at a density of 550 cells/mm^2^ on pre-treated substrates. Primary cortical cells were plated on the plating medium and allowed to adhere for 4 h before the medium was changed to the co- or tri-culture medium. Half-media changes were performed at day in vitro (DIV) 3 and 7 with the respective media types.

### 2.2. TLR Agonist Treatment

The TLR agonists used were as follows (all purchased from InvivoGen, San Diego, CA, USA): Pam3CSK4 (TLR1/2 agonist), heat-killed *Listeria monocytogenes* (HKLM, TLR2 agonist), Poly(I:C) both high molecular weight (HMW) and low molecular weight (LMW, TLR3 agonist), lipopolysaccharide (LPS, TLR4 agonist), flagellin from *Salmonella typhimurium* (FLA-ST, TLR5 agonist), FSL1 (TLR6/2 agonist), R848 (Resiquimod, TLR7/8 agonist), and ODN1826 (TLR9 agonist). Prior to use, all ligands were reconstituted in sterile DI water at 200× (the maximum working concentration) and stored at −20 °C. To spike the cultures, at DIV 7, half of the medium was removed from each well and replaced with an equal volume of medium that contained a 2× working concentration of a TLR agonist or an equal volume (~1/200 of the medium volume) of sterile DI water to act as the vehicle control. The cultures were then maintained for 48 h, after which they were analyzed with one of the following methods.

### 2.3. Immunostaining

At the conclusion of the experiment, the cell cultures were washed three times with 37 °C DPBS+ and fixed using 4% *w*/*v* paraformaldehyde (PFA; Affymetrix, Santa Clara, CA, USA) in PBS for 2.5 h. Fixed cells were washed twice with 0.05% *v*/*v* Tween20 (Sigma) solution in DPBS+, followed by a 3 min permeabilization with 0.1% *v*/*v* Triton X-100 (ThermoFisher) solution in DPBS+ and two additional washes with Tween20 solution. Samples were blocked with a solution of 0.5% *v*/*v* heat-inactivated goat serum (ThermoFisher) and 0.3 M glycine (Sigma) in DPBS+ (blocking buffer) for 1 h. Following the blocking step, samples were incubated for 1 h in a primary antibody solution containing mouse anti-βIII tubulin (ThermoFisher), rabbit anti-GFAP (ThermoFisher), and chicken anti-Iba1 (Abcam, Cambridge, UK) in blocking buffer. Samples were then washed three times with Tween20 solution before a 1 h incubation with secondary antibody solution containing goat anti-mouse antibodies conjugated to AlexaFluor 647 (ThermoFisher), goat anti-rabbit antibodies conjugated to AlexaFluor 488 (ThermoFisher), and goat anti-chicken antibodies conjugated to AlexaFluor 555 (ThermoFisher). Following incubation with secondary antibody solution, the samples were washed three times with DPBS+. Lastly, samples were incubated for 5 min with a 4′,6-diamidino-2-phenylindole (DAPI) solution (Sigma) to stain cell nuclei, followed by an additional Tween20 solution wash before they were mounted onto glass slides using ProLong Gold Antifade Mountant (ThermoFisher).

### 2.4. Live/Dead, Apoptosis, and Morphological Analysis

Following the 48 h exposure to TLR agonists, each well was incubated with both Hoechst 33342 (ThermoFisher) and propidium iodide (ThermoFisher) directly added to the medium to yield working concentrations of 1 µg/mL and 1 µM, respectively, and incubated for 20 min at room temperature. Apoptotic cells were quantified via annexin V staining using the ab176749 Apoptosis/Necrosis Assay Kit (Abcam) according to the manufacturer’s protocol. For morphological analysis, cultures were fixed with a 4% *w*/*v* PFA solution in PBS and immunostained as described above. All sample images were acquired with a Zeiss Observer D1 inverted epi-fluorescence microscope at 100× or 200× magnification and analyzed using ImageJ. The number of live/dead cells and percentage of area positive for annexin V staining were quantified using custom ImageJ macros [25]. The average astrocyte/microglia areas were determined by manually tracing the outline of astrocytes/microglia from 200× magnification images and determining the area inside the trace (representative examples are shown in Figure S1).

### 2.5. Statistical Methods

For all experiments requiring image analysis, at least five predetermined fields were analyzed per replicate to account for variability within the culture itself. All data was analyzed using a one-way analysis of variance (ANOVA) test. Differences between groups were identified by a post hoc Tukey test or Dunnett’s test. For all experiments, a *p*-value < 0.05 was considered statistically significant and all data is presented as mean ± standard error of the mean (SEM). All statistical analyses were performed with R.

## 3. Results

### 3.1. Influence of Microglia Presence on Cell Death

**Figure 1:**
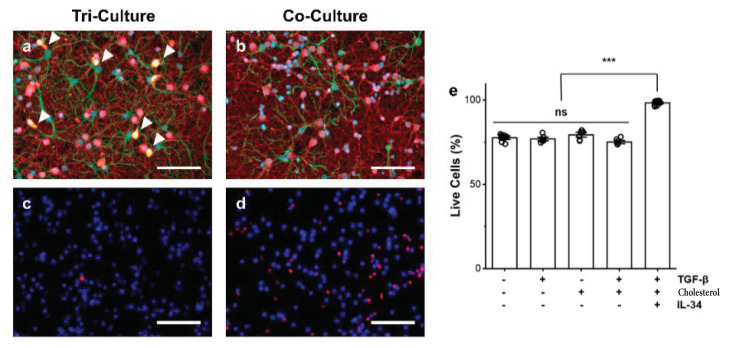
Representative fluorescence images of the (**a**) tri-culture and (**b**) co-culture at DIV 9 demonstrating the presence of microglia in the tri-culture. The cultures were immunostained for the three cell types of interest: neurons—anti-βIII-tubulin (red), astrocytes—anti-GFAP (green), microglia—anti-Iba1 (orange), general nuclear stain DAPI (blue). As expected, microglia are only present in the tri-culture (highlighted with arrows). Representative images of (**c**) tri-cultures and (**d**) co-cultures stained with Hoechst 33342 (Live, blue) and propidium iodide (Dead, red). (**e**) Comparison of the influence of TGF-β and increased cholesterol concentration on cell death in the absence of microglia as compared to the co-culture and tri-culture (containing all three additional factors). Data presented as mean ± SEM (n = 4–8 wells from two independent dissections) with individual data points plotted, *** *p* < 0.001 (as determined by post hoc Tukey’s test). Scale bar = 100 µm.

We have previously demonstrated that there is an improvement in the overall health of neuron, astrocyte, and microglia tri-cultures as compared to neuron–astrocyte co-culture due to the continuous presence of microglia [21]. As expected, we observed a physiologically relevant population of microglia (Figure 1a) that was not present in the neuron–astrocyte co-culture (Figure 1b). Additionally, we wanted to ensure that the presence of the additional factors added to the tri-culture medium in the absence of microglia did not affect the overall health of the culture. Therefore, we compared the total cell death of the co-culture (Figure 1d) to both the tri-culture (Figure 1c) and cultures maintained in the co-culture medium supplemented with TGF-β, cholesterol, or both factors at concentrations used in the tri-culture medium (Figure 1e). We did not compare conditions with IL-34 as we have previously shown that IL-34 alone is sufficient and necessary for microglia survival in vitro [21], and the present goal was to determine if the additional factors in the tri-culture media (TGF-β and cholesterol) in the absence of microglia had an effect on culture health. As expected, we saw a significant reduction in cell death in the tri-culture (1.63 ± 0.32% dead cells) as compared to the co-culture (22.33 ± 0.44% dead cells, *p* < 1.0 × 10^−7^). Additionally, there was no change in cell death when TGF-β (23.06 ± 0.86% dead cells), cholesterol (20.89 ± 1.10% dead cells), or both factors (24.46 ± 0.67% dead cells) were added to the co-culture medium, which all showed a significant increase in cell death as compared to the tri-culture (*p* < 1.0 × 10^−7^ for all conditions vs. tri-culture).

### 3.2. Influence of TLR Agonists on Cell Death

**Figure 2:**
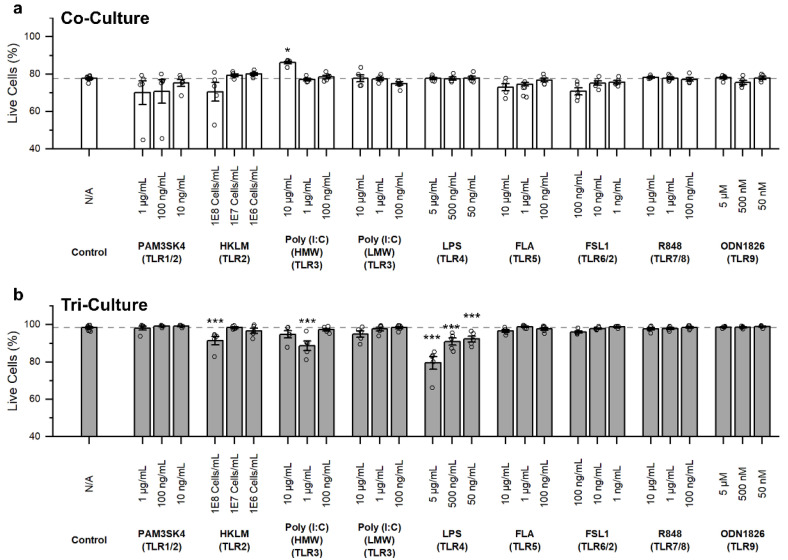
Influence of TLR agonist treatment on cell death as quantified by staining the cultures with Hoechst 33342 (Live) and propidium iodide (Dead). The overall percentage of live cells in (**a**) co-cultures and (**b**) tri-cultures following TLR agonist treatment. Data shown as mean ± SEM (n = 6 wells from two independent dissections) with individual data points plotted, * *p* < 0.05, *** *p* < 0.001 (as determined by a one-way ANOVA followed by Dunnett’s test vs. vehicle control).

In order to determine the role of microglia on the overall neuroinflammatory impact of TLR activation, we compared the response of both the co- and tri-cultures to an array of known TLR agonists at a range of concentrations (Figure 2). These concentrations were individually chosen for each TLR agonist based on concentrations that have been shown to elicit a response from immune cells and covered two orders of magnitude [21,26,27,28,29,30,31]. As expected, we observed a significant increase in cell death in the tri-culture following a 48 h incubation with LPS (TLR4 agonist) at all concentrations (*p* < 2 × 10^−16^, *p* = 1.1 × 10^−5^, and *p* = 6.6 × 10^−4^ when incubated with 5 µg/mL LPS, 500 ng/mL and 50 ng/mL, respectively) as compared to the vehicle, which was not observed in the co-culture (*p* = 1.0 for all concentrations). We also observed a significant increase in cell death following treatment with the highest concentration of HKLM (1 × 10^8^ cells/mL, TLR2 agonist) in the tri-culture (*p* = 9.04 × 10^−4^), and while treatment with 1 × 10^8^ cells/mL HKLM did show an increase in cell death in the co-culture, this increase did not prove to be statistically significant (*p* = 0.10). Interestingly, we saw a non-monotonic relationship between HMW poly (I:C) (TLR3) treatment concentration and cell death, where treatment with 1 µg/mL HMW poly (I:C) induced a significant increase in cell death (*p* = 5.45 × 10^−9^), while the 10 µg/mL and 100 ng/mL treatment concentrations did not result in cell death (*p* = 0.21 & 1.0, respectively). For the co-culture, treatment with 10 µg/mL HMW poly (I:C) led to a significant reduction in cell death (*p* = 0.023), which was the only instance of a reduction of cell death across all conditions. Additionally, for the co-culture there were trends towards increased cell death following treatment with 100 ng/mL Pam3SK4 (TLR1/2 agonist, *p* = 0.066), 1 × 10^8^ cells/mL HKLM (TLR2 agonist, *p* = 0.10), and 100 ng/mL FSL1 (TLR6/2 agonist, *p* = 0.14).

### 3.3. Influence of TLR Agonists on Apoptosis

**Figure 3:**
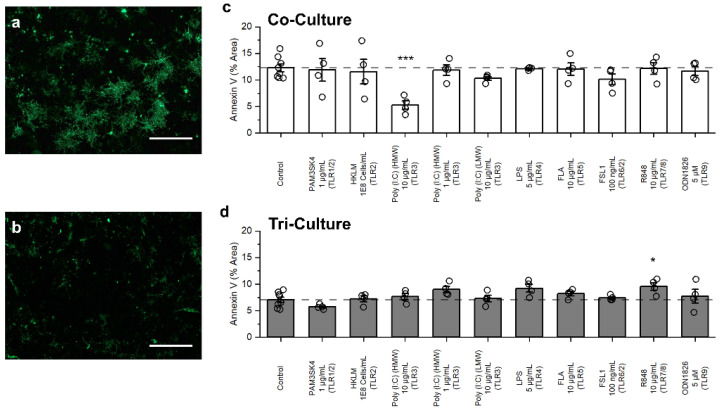
Influence of TLR agonist treatment on Annexin V staining. Representative fluorescence images of the (**a**) co-culture and (**b**) tri-culture at DIV 9. Quantification of the Annexin V staining in the (**c**) co-cultures and (**d**) tri-cultures following 48 h exposure to TLR agonists. Data presented as mean ± SEM (n = 4 wells from two independent dissections) with individual data points plotted, * *p* < 0.05, *** *p* < 0.001 (as determined by a one-way ANOVA followed by Dunnett’s test vs. vehicle control). Scale bar = 100 µm.

Motivated by the influence of TLR agonist treatment on cell death in both the co- and tri-culture configurations, we studied whether TLR agonist treatment induced other, potentially subtle, changes in the cultures. Therefore, we compared the amount of Annexin V staining (a marker for apoptosis) in both the co- and tri-cultures in response to TLR agonist treatments. Specifically, we treated the cultures with the highest concentration of each TLR agonists along with 1 µg/mL HMW poly (I:C) as we observed a difference in cell death in the tri-culture at this concentration that was not observed at the higher concentration. We observed significantly more Annexin V staining in the control conditions of the co-culture (12.27 ± 0.72%) as compared to the tri-culture (7.05 ± 0.51%, *p* = 5.61 × 10^−5^) (Figure 3a,b) in agreement with our previous study which used caspase 3/7 activity to quantify apoptosis [21]. Similar to cell death, we observed a significant decrease in the Annexin V staining in the co-culture treated with the 10 µg/mL HMW poly (I:C) (TLR3 agonist) as compared to the control (*p* = 3.55 × 10^−4^, Figure 3c) that was not observed in the tri-culture (*p* = 0.99, Figure 3d). For the tri-culture we observed a significant increase in Annexin V staining following treatment with 10 µg/mL R848 (TLR7/8 agonist, *p* = 0.036, Figure 3d). Additionally, we saw a trend towards increased Annexin V staining following treatment with 5 µg/mL LPS (TLR4 agonist, *p* = 0.094) and 1 µg/mL HMW poly (I:C) (*p* = 0.17). Overall, we observed that compared to the co-culture, the tri-culture showed a more robust an increase in AnnexinV staining in response to TLR agonist treatment (Figure S2).

### 3.4. Influence of TLR Agonists on Glial Cell Morphology

**Figure 4:**
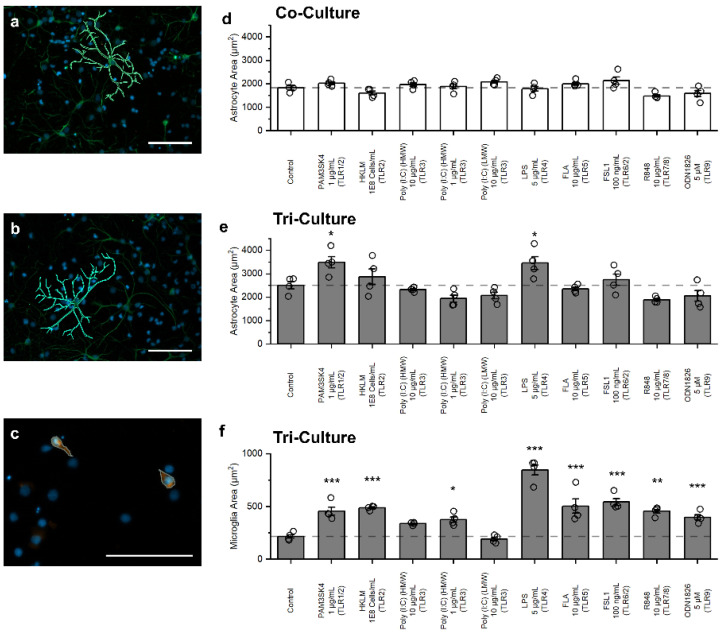
Influence on TLR agonist treatment on glial cell morphology. Representative fluorescence images of astrocytes in the (**a**) co-culture and (**b**) tri-culture, and (**c**) microglia in the tri-culture at DIV 9 showing the outlining method used to quantify area. Quantification of the average astrocyte area in (**d**) co-cultures and (**e**) tri-cultures following 48 h exposure to TLR agonists. (**f**) Quantification of the average microglia area in the tri-cultures following 48 h exposure to TLR agonists. Data presented as mean ± SEM (n = 4 wells from two independent dissections) with individual data points plotted, * *p* < 0.05, ** *p* < 0.01, *** *p* < 0.001 (as determined by a one-way ANOVA followed by Dunnett’s test vs. vehicle control). Scale bar = 100 µm.

Reactive astrocytes, typically characterized by a hypertrophic morphology, are often used as markers of neuroinflammation in neurodegenerative [32] and autoimmune disorders [33]. Therefore, we quantified the change in astrocyte area in both the co- and tri-cultures in response to TLR agonist treatment (Figure 4a–c). Upon exposure to TLR agonists, we did not observe any significant changes to astrocyte area in the co-cultures (Figure 4d). However, for the tri-culture, significant astrocyte hypertrophy was evident following treatment with 1 µg/mL PAM3CSK4 (*p* = 0.033) and 5 µg/mL LPS (*p* = 0.044) (Figure 4e). Change to microglia morphology is another common marker used to identify neuroinflammation, and we have previously shown that in culture, microglia adopt a spread morphology in response to neuroinflammatory stimuli [21]. Therefore, we also quantified the change in microglia morphology in the tri-culture following TLR agonist treatment. We observed a significant increase in microglia area for every treatment condition with the exception of 10 µg/mL HMW poly (I:C) and 10 µg/mL LMW poly (I:C) (Figure 4f).

## 4. Discussion

In vitro methods are powerful tools for studying the specific cellular and molecular mechanisms behind many neurological and neurodegenerative diseases [34,35]. However, they are also limited in capturing the in vivo microenvironment that is composed of complex physical and biochemical cues. One way to improve in vivo relevance of an in vitro model (e.g., cell culture) is to incorporate critical cell types to maintain cell-to-cell communication, such as including neurons and glial cells in the context of cortical neural tissue, to better mimic inflammation and other pathological states [36]. To that end, we studied the influence of the neural cell culture’s composition on its neuroinflammatory response to an array of TLR agonists. We specifically revealed the important role of microglia by using rodent primary cortical cell cultures with and without microglia. Additionally, we demonstrated the significant benefit of the tri-culture model’s relative simplicity for use in screening large numbers of treatments.

In our previous work, we demonstrated that the continuous presence of microglia in a tri-culture of neurons, astrocytes, and microglia did not negatively affect the overall health of the culture and resulted in decreased caspase 3/7 activity (a marker for apoptosis) [21]. Here, we further show that the presence of viable and functional microglia significantly increases the percentage of live cells in the culture as compared to a sole neuron–astrocyte co-culture, and that this increase is mediated by the presence of microglia (dictated by the presence of IL-34 [37,38,39]) and not by the additional factors (i.e., TGF-β or cholesterol alone) present in the tri-culture medium required to maintain physiologically active microglia (Figure 1). This confirms that the improved culture health is primarily due to cross-talk between microglia, neurons, and astrocytes, and changes the cytokine profile of the culture as discussed in our previous publication [21].

Previous work has shown that microglia from mice [22] and humans [20] constitutively express mRNA for TLR1–9, and our results suggest that rat microglia also constitutively express TLR1–9. Specifically, following 48 h exposure to TLR agonists, we observed a significant increase in microglia area, indicative of microglia activation [40,41,42,43] for all TLR agonists with the exception of LMW poly(I:C) (Figure 4f). However, both LMW poly(I:C) and HMW poly(I:C) activate TLR3, indicating that microglia respond to TLR3 agonists, but only at specific concentrations, as it has been shown that LMW poly(I:C) has a much lower activation efficiency for TLR3 as compared to HMW poly(I:C) [44]. Additionally, while we observed morphological changes in response to TLR agonist treatments, not all treatments induced further indicators of neuroinflammation such as changes to cell death or apoptosis. Conversely, while it has been suggested that astrocytes express mRNA for TLR1–9 [15,45,46], it has been shown that astrocytes express mRNA for TLR3 at much higher levels than the other TLRs [20]. Similarly, when quantifying the different aspects of neuroinflammation in the co-culture lacking microglia, we only observed changes to cell death and apoptosis in response to TLR3 activation, with no other TLR ligands inducing changes to cell death, apoptosis, or astrocyte morphology. Therefore, while we have not directly quantified the expression levels of different TLRs in the astrocytes and microglia in our cultures, the results from this study suggest that conclusions drawn from mouse and human studies can be extended to our primary rat tri-culture model.

Of all the TLR agonists tested, FLA (TLR5 agonist), FSL1 (TLR6/2 agonist), and ODN1826 (TLR9 agonist) did not show any additional indicators of neuroinflammation in either the co- or tri-cultures beyond changes to microglia morphology. Compared to other TLRs, the role of TLR5 activation in relationship to neuroinflammation and other neurological diseases has received less attention, with much of the research focusing on the expression levels of TLR5 in different CNS cell types and not on the role TLR5 activation may have on neuroinflammation [17,46]. One recent study has shown that activation of TLR5 on microglia induces neural cell loss and apoptosis using a microglia and neuron co-culture [47]. Interestingly, we did not observe the same neurotoxic effects although we used the same source and concentration of TLR5 agonists for our treatment. One likely cause of this discrepancy may be the difference in the culture methodology, including animal model (rat vs. mouse), age (perinatal vs. embryonic), and culture composition (neuron–microglia co-culture vs. tri-culture). Another potential reason for the increased neurotoxic features observed by Ifuku et al. is that the isolated microglia culture used in their study contained 10% fetal bovine serum (FBS), which has been shown to activate microglia [48], and may be working in combination with the TLR5 activation to produce a more pronounced neurotoxic effect. Activation of TLR9 on microglia has also been shown to induce significant neuronal death in vivo [49] that was not observed in our tri-culture. However, this cell death was primarily attributed to the breakdown of the blood brain barrier (BBB) [49], and therefore it is not surprising that the tri-culture was unable to capture this effect.

Activation of TLR4 in microglia by LPS is a well characterized method to induce robust neurotoxic neuroinflammation both in vitro [50] and in vivo [51]. The results from this study are in line with our previous work showing significant increases in apoptosis and astrocyte and microglia hypertrophy in the tri-culture following exposure to LPS [21]. While we do not observe a statistically significant increase in AnnexinV staining in this study, the relative change of AnnexinV staining vs. control is similar in magnitude to the increase in caspase 3/7 activity in our previous study, and thus the lack of statistical significance in this study (*p* = 0.094) may be a function of the need to control for the familywise error rate (in the case of multiple comparisons). In addition, we demonstrate a significant increase in cell death in the tri-culture following 48 h exposure to LPS at concentrations as low as 50 ng/mL confirming that the tri-culture can replicate the robust neuroinflammatory response to LPS treatment seen in vivo. Similarly, the response of the co-culture is as expected with no changes to cell death, AnnexinV staining, or astrocyte morphology, as the culture lacks microglia.

TLR2 has a wide spectrum capable of recognizing a variety of PAMPs [52], and TLR1 and 6 heterodimerize with TLR2 to further enhance the recognition of different PAMPs. The heterodimerization has been attributed to an evolutionary mechanism of expanding the ligand recognition spectrum of various cell types to the diverse lipopeptide structure of different pathogens [53]. In this study, TLR2 was activated with HKLM, which is a well-known agonist that is used both for in vivo [54] and in vitro [55,56] studies. Along with TLR2, the TLR1, and TLR6 heterodimers were activated with PAM3CSK4 and FSL-1 additions to the culture, respectively. We observed an increasing trend of cell death in the co-culture with TLR1/2, 2 and 6/2 agonists, whereas the cell death in the corresponding tri-culture groups was negligible except for in the tri-culture group treated with the highest concentration of the TLR2 agonist. Surprisingly, while cell death was not significant for the tri-culture group treated with TLR1/2 agonist, there was significant astrocyte hypertrophy for this group only (in contrast to the groups treated with TLR 2 and 6/2 agonists). The underlying reasons for these seemingly contradictory phenomena require mechanistic studies and may be due to the dual effects (anti-inflammatory vs. pro-inflammatory) of some cytokines secreted as function of TLR activation (e.g., IL-6) [57] and the cooperative response of these TLRs exhibiting non-monotonous dependence on agonist concentrations. Importantly, these results highlight that the presence of microglia reveals complex responses compared to those from neuron–astrocyte co-cultures.

The opposite responses of the co- and tri-cultures to exposure to TLR3 agonist highlights the importance of culture composition on the observed outcomes of neuroinflammatory stimuli. The neuron–astrocyte co-culture shows a significant decrease in cell death and AnnexinV staining in following 48 h exposure to 10 µg/mL HMW poly(I:C). This is in line with previous reports showing that isolated astrocyte cultures produce neuroprotective cytokines in response to TLR3 activation, and that conditioned media from poly(I:C)-treated purified astrocyte cultures improved neuron survival in organotypic cultures [19]. Conversely, it has been shown that activation of microglial TLR3 leads to increased production of the pro-inflammatory cytokines TNF-α and IL-6 [58]. Interestingly, the tri-culture was able to capture both the neuroprotective aspect of TLR3 activation on astrocytes and the more neurotoxic aspect of microglia TLR3 activation leading to a non-monotonic change in cell death in response to HMW poly(I:C) concentration in the tri-culture. We observed a significant increase in cell death following treatment with 1 µg/mL HMW poly(I:C), which was not observed at higher (10 µg/mL) treatment concentrations, putatively due to the neuroprotective aspects of astrocyte TLR3 activation counteracting the neurotoxic aspects of microglia TLR3 activation.

We did not observe a significant change in cell death or astrocyte morphology in either the co- or tri-culture following exposure to R848 (TLR7/8 agonist). However, in the tri-culture we did see a significant increase in AnnexinV staining that was not seen in the co-culture. While there has not been much research into the impact of astrocyte TLR7/8 activation [17,46], it has been shown that activation of TLR8 in primary microglia cultures from non-human primates leads to an increase in production of pro-inflammatory cytokines such as TNF-α and IL-12 [59]. Additionally, knocking-down (KD) of TLR7 in mice lead to a reduction in the production of IL-6 following infections with Japanese encephalitis virus (JEV); however, these mice also showed increased viral load and mortality [60]. It has also been shown that R848 treatment can directly interact with TLR8 receptors on neurons, leading to reduced neurite outgrowth and increased apoptosis [61]. As we did not observe an increase in apoptosis in the co-culture, it is unlikely that direct activation of TLR8 on the neurons was solely responsible for the increase in AnnexinV staining in the tri-culture. It is most likely that the increase in proinflammatory factors driven by the activation of microglia TLR7/8, or a combination of the two effects, is responsible for the increased apoptosis observed in the tri-culture.

## 5. Conclusions

In this study, we demonstrated the importance of microglia on the observed neuroinflammatory response to TLR activation in primary cortical cultures (summarized in Table 1). In response to TLR2, 3, 4, and 7/8, we observed significant neurotoxic responses including increases in cell death and apoptosis in tri-cultures of neurons, astrocytes, and microglia that were not observed in co-cultures of only neurons and astrocytes. The only significant change that we observed in the co-culture was a decrease in cell death and apoptosis in response to TLR3 activation. Future studies comparing changes in cytokine production would further the understanding of the role microglia play in the neuroinflammatory response to TLR activation. Additionally, comparing the co-activation of different TLRs may also reveal differences in the neuroinflammatory response, as previous work has shown unique neuroinflammatory responses to different pairwise combinations of TLR agonists [62]. Cell culture models are vital tools for gaining a better understanding of the cellular and molecular processes underlying many neurological and neurodegenerative disorders. However, it is also critical to recognize the inherent limitations within these models. The use of mixed culture models, such as the tri-culture used in this study, is one method of overcoming some of these limitations. By including neurons, astrocytes, and microglia in a single culture model, we were able to observe the relative influence of all three cell types on the neuroinflammatory response to different TLR agonists. This was particularly apparent in the response to TLR3 activation, in which we observed that microglia and astrocytes play opposing roles in the neuroinflammatory response and depending on the concentration of TLR3 agonist the response of microglia or astrocytes dominated the observed response. Additionally, as the only modification required to establish and maintain the tri-culture is the preparation and use of the specific tri-culture medium, we demonstrate that the tri-culture is well suited for higher-throughput experiments. Overall, we have demonstrated that microglia play a significant role in the neuroinflammatory response to TLR activation in vitro, and their presence is critical to better replicate the in vivo response. Furthermore, we expect that the tri-culture model and the accompanying methods described here will be useful for higher-throughput screening studies that range from neurotoxins to pharmaceuticals.

## Figures and Tables

**Table 1:** Summary of co- and tri-culture response to TLR activation as compared to their respective vehicle controls.

Toll-like Receptor Activation	Co-Culture	Tri-Culture
TLR1/2	No Change	Increased Astrocyte Area Increased Microglia Area
TLR2	No Change	Increased Cell Death Increased Microglia Area
TLR3	Reduced Cell Death Reduced Apoptosis	Increased Cell Death ^1^ Increased Microglia Area ^1^
TLR4	No Change	Increased Cell Death Increased Astrocyte Area Increased Microglia Area
TLR5	No Change	Increased Microglia Area
TLR6/2	No Change	Increased Microglia Area
TLR7/8	No Change	Increased Apoptosis Increased Microglia Area
TLR9	No Change	Increased Microglia Area

^1^ Dependent on ligand concentration.

## Data Availability

The data presented in this study are available on request from the corresponding author.

## Supplementary Materials

The following supporting information can be downloaded at: https://www.mdpi.com/article/10.3390/biomedicines10092122/s1, Figure S1: Representative fluorescence images of glia morphology of all TLR agonist treatments; Figure S2: Comparison of the change in the number of live cells, AnnexinV staining, and astrocyte area between the co- and tri-cultures following TLR agonist treatment.
