# Supplementary material for: Primary Cortical Cell Tri-Culture-Based Screening of Neuroinflammatory Response in Toll-like Receptor Activation
Source: Biomedicines. 2022 Aug 29;10(9):2122. doi: 10.3390/biomedicines10092122 (PMC9495748; doi:10.3390/biomedicines10092122)
Supplement: Supplementary file 1 [file biomedicines-10-02122-s001.zip › biomedicines-1868424-supplementary.pdf]

biomedicines

MDPI

Supplementary Material

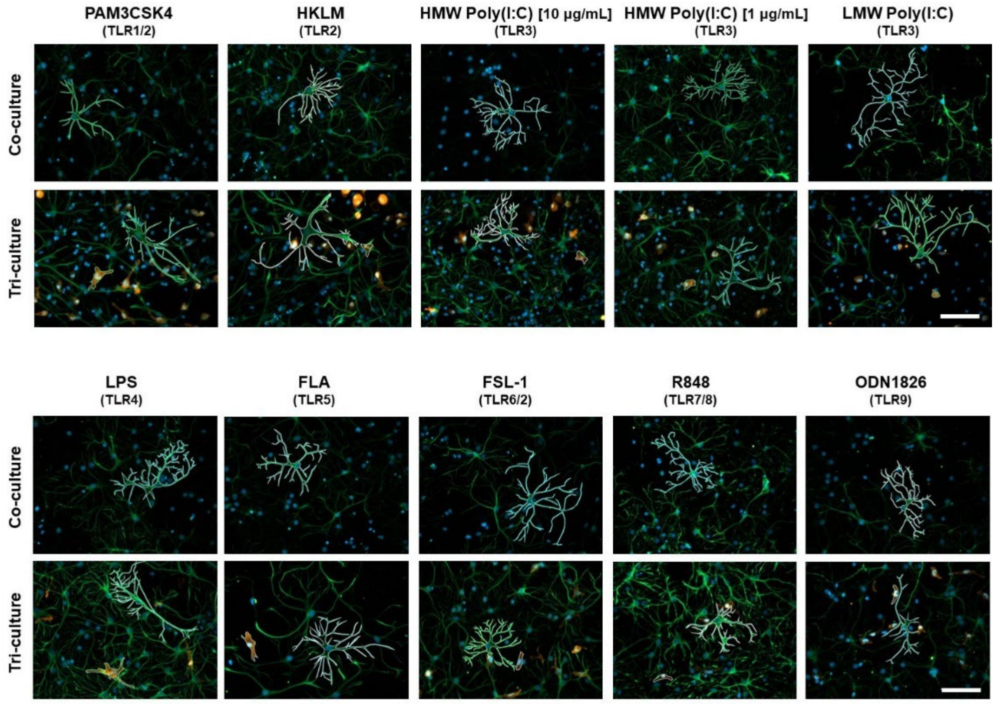

Figure S1. Representative fluorescence images of glia morphology of all TLR agonist treatments showing the outlining method used to quantify glia area. The cultures were immunostained for the two glial cell types: astrocytes—anti-GFAP (green), microglia—anti-Iba1 (orange), and general nuclear stain DAPI (blue). Scale bar = 100 µm.

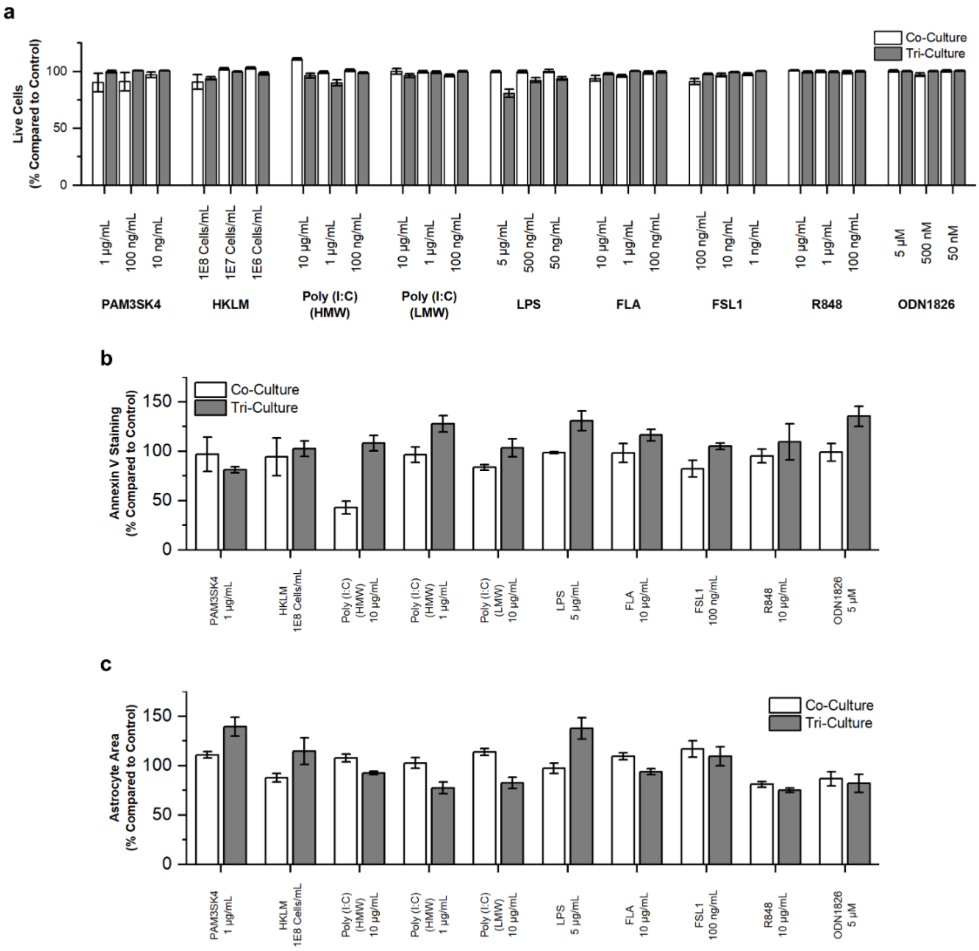

Figure S2. Comparison of the change in the number of (**a**) live cells, (**b**) AnnexinV staining, and (**c**) astrocyte area between the co- and tri-cultures following TLR agonist treatment normalized to vehicle control.
